# Supplementary material for: Associations between maternal iron supplementation in pregnancy and offspring growth and cardiometabolic risk outcomes in infancy and childhood
Source: PLoS One. 2022 May 27;17(5):e0263148. doi: 10.1371/journal.pone.0263148 (PMC9140278; doi:10.1371/journal.pone.0263148)
Supplement: S1 Table — (DOCX) [file pone.0263148.s001.docx]

**S1 Table** Characteristics of those participants who took part in both the infancy and follow-up study and those participants that only took part in the infancy studies.

| **Clinical characteristic** | **Infancy Study Alone** | **Infancy & Mid‑Childhood Studies** | **p‑value** |
| --- | --- | --- | --- |
| Mother supplemented their diet with iron in pregnancy (n yes/no) | 471/313 | 122/79 | 0.9 |
| Gestational age at birth (weeks) | 39.9  (39.7, 40.0)  (n=767) | 39.9  (39.7, 40.2)  (n=201) | 0.5 |
| Birth weight (kg) ^1^ | 3.510  (3.478, 3.542)  (n=763) | 3.523  (3.460, 3.585)  (n=201) | 0.7 |
| Birth length (cm) ^2^ | 51.5  (51.4, 51.7)  (n=744) | 51.5  (51.3, 51.8)  (n=194) | 0.9 |
| Head circumference at birth (cm) ^2^ | 35.4  (35.3, 35.5)  (n=744) | 35.2  (35.1, 35.4)  (n=196) | 0.1 |
| Ponderal index at birth (kg/m^3^) ^2^ | 25.6  (25.4, 25.8)  (n=742) | 25.8  (25.5, 26.2)  (n=194) | 0.3 |
| Sex (n males/females) | 417/348 | 88/113 | 0.007 |
| Mother smoked in pregnancy (n yes/no) | 28/738 | 3/198 | 0.1 |

Data are numbers of CBGS participants or means (95% confidence intervals).

^1^adjusted for gestational age at birth and sex.

^2^adjusted for gestational age at birth, age at assessment and sex.
