# Supplementary material for: Associations between maternal iron supplementation in pregnancy and offspring growth and cardiometabolic risk outcomes in infancy and childhood
Source: PLoS One. 2022 May 27;17(5):e0263148. doi: 10.1371/journal.pone.0263148 (PMC9140278; doi:10.1371/journal.pone.0263148)
Supplement: S2 Table — (DOCX) [file pone.0263148.s002.docx]

**S2 Table** Associations between maternal iron supplementation status in pregnancy and markers of offspring growth at 3 months of age.

| **Measurement** | **No Maternal Iron Supplementation in Pregnancy** | **Maternal Iron Supplementation in Pregnancy** | **p‑value** |
| --- | --- | --- | --- |
| Weight (kg) | 6.1  (6.0, 6.2)  (n=363) | 6.2  (6.1, 6.2)  (n=534) | 0.1 |
| Height (cm) | 61.0  (60.7, 61.2)  (n=359) | 61.2  (61.0, 61.4)  (n=534) | 0.1 |
| Head circumference (cm) | 40.8  (40.6, 40.9)  (n=362) | 40.8  (40.7, 40.9)  (n=535) | 1.0 |
| Body mass index (kg/m^2^) | 16.4  (16.2, 16.5)  (n=359) | 16.5  (16.4, 16.6)  (n=532) | 0.4 |

Data are mean (95% confidence interval) adjusted for age and sex.
