# Supplementary material for: Associations between maternal iron supplementation in pregnancy and offspring growth and cardiometabolic risk outcomes in infancy and childhood
Source: PLoS One. 2022 May 27;17(5):e0263148. doi: 10.1371/journal.pone.0263148 (PMC9140278; doi:10.1371/journal.pone.0263148)
Supplement: S3 Table — (DOCX) [file pone.0263148.s003.docx]

**S3 Table** Associations between maternal iron supplementation status in pregnancy and offspring growth and physiological measurements at around age 9.5 years in those children whose mothers did not report being anaemic in pregnancy.

| **Childhood Measurement** | **No Maternal Iron Supplementation in Pregnancy** | **Maternal Iron Supplementation in Pregnancy** | **p‑value** |
| --- | --- | --- | --- |
| Weight (kg)^1^ | 32.3  (31.1, 33.6)  (n=75) | 32.1  (31.1, 33.1)  (n=109) | 0.8 |
| Height (m)^1^ | 1.39  (1.38, 1.40)  (n=74) | 1.39  (1.38, 1.40)  (n=110) | 0.9 |
| Head circumference (cm)^1^ | 53.3  (53.0, 53.6)  (n=75) | 53.1  (52.9, 53.4)  (n=110) | 0.3 |
| Waist circumference (cm)^1^ | 59.7  (58.1, 61.3)  (n=72) | 59.6  (58.3, 60.9)  (n=107) | 0.9 |
| Flank skinfold thickness (mm)^1^ | 10.4  (9.5, 11.3)  (n=75) | 11.0  (10.2, 11.8)  (n=110) | 0.3 |
| Quadriceps skinfold thickness (mm)^1^ | 15.5  (14.4, 16.7)  (n=74) | 16.1  (15.1, 17.1)  (n=106) | 0.5 |
| Subscapular skinfold thickness (mm)^1^ | 7.0  (6.5, 7.6)  (n=75) | 6.9  (6.4, 7.4)  (n=110) | 0.7 |
| Triceps skinfold thickness (mm)^1^ | 10.3  (9.6, 11.1)  (n=75) | 10.9  (10.2, 11.6)  (n=110) | 0.3 |
| Systolic blood pressure (mmHg)^2^ | 106.5  (104.0, 109.0)  (n=70) | 103.8  (101.8, 105.8)  (n=103) | 0.1 |
| Diastolic blood pressure (mmHg)^2^ | 62.0  (60.4, 63.6)  (n=70) | 59.9  (58.6, 61.2)  (n=103) | 0.04 |
| Mean arterial blood pressure (mmHg)^2^ | 76.3  (74.6, 78.0)  (n=70) | 74.0  (72.7, 75.4)  (n=103) | 0.04 |
| Pulse pressure (mmHg)^2^ | 43.8  (41.8, 45.9)  (n=70) | 43.1  (41.5, 44.8)  (n=103) | 0.6 |
| Pulse (/min)^2^ | 75.8  (73.3, 78.4)  (n=60) | 74.9  (72.7, 77.2)  (n=74) | 0.6 |
| Fasting glucose concentration (mmol/L)^1^ | 5.0  (4.9, 5.1)  (n=54) | 5.0  (4.9, 5.1)  (n=93) | 0.6 |
| OGTT 30 min. glucose concentration (mmol/L)^1^ | 8.3  (7.9, 8.7)  (n=54) | 8.2  (7.9, 8.5)  (n=94) | 0.6 |
| HOMA IR^1^ | 0.908  (0.769, 1.072)  (n=53) | 0.793  (0.689, 0.913)  (n=75) | 0.2 |
| Fasting C‑peptide concentration (nmol/L)^1^ | 292  (265, 322)  (n=54) | 272  (252, 293)  (n=94) | 0.3 |
| OGTT insulin increment (pmol/L)^1^ | 328  (274, 393)  (n=49) | 294  (257, 338)  (n=87) | 0.3 |
| OGTT insulinogenic index^1^ | 108  (91, 129)  (n=48) | 97  (85, 111)  (n=84) | 0.3 |
| OGTT insulin disposition index^1^ | 4.28  (3.59, 5.10)  (n=48) | 4.18  (3.66, 4.78)  (n=84) | 0.8 |

Data are mean (95% confidence interval).

^1^Adjusted for age and sex.

^2^Adjusted for age, sex and arm of measurement.
